# Dynamic Modeling of Cost-effectiveness of Rotavirus Vaccination, Kazakhstan

**DOI:** 10.3201/eid2001.130019

**Published:** 2014-01

**Authors:** Birgitte Freiesleben de Blasio, Elmira Flem, Renat Latipov, Ajnagul Kuatbaeva, Ivar Sønbø Kristiansen

**Affiliations:** Norwegian Institute of Public Health, Oslo, Norway (B. Freiesleben de Blasio, E. Flem);; University of Oslo, Norway (B. Freiesleben de Blasio, I.S. Kristiansen);; Research Institute of Virology, Tashkent, Republic of Uzbekistan (R. Latipov);; Scientific-Practical Centre of Epidemiologic Surveillance, Almaty, Republic of Kazakhstan (A. Kuatbaeva)

**Keywords:** viruses, rotavirus, vaccination, cost-effectiveness, dynamic modeling, herd immunity, break-even price, vaccine, Kazakhstan

## Abstract

The government of Kazakhstan, a middle-income country in Central Asia, is considering the introduction of rotavirus vaccination into its national immunization program. We performed a cost-effectiveness analysis of rotavirus vaccination spanning 20 years by using a synthesis of dynamic transmission models accounting for herd protection. We found that a vaccination program with 90% coverage would prevent ≈880 rotavirus deaths and save an average of 54,784 life-years for children <5 years of age. Indirect protection accounted for 40% and 60% reduction in severe and mild rotavirus gastroenteritis, respectively. Cost per life year gained was US $18,044 from a societal perspective and US $23,892 from a health care perspective. Comparing the 2 key parameters of cost-effectiveness, mortality rates and vaccine cost at <US $2.78 per dose, vaccination program costs would be entirely offset. To further evaluate efficacy of a vaccine program, benefits of indirect protection conferred by vaccination warrant further study.

## Abstract 2

Comparison of projected rotavirus vaccine health effects and costs over 20 years showed its use to be cost-effective.

Cost-effectiveness of Rotavirus Vaccination Program

Rotavirus is the leading cause of severe acute gastroenteritis in children worldwide (*1*). Rotavirus vaccines Rotarix (GlaxoSmithKline Biologicals, Rixensart, Belgium) and Rotateq (Merck & Co., Whitehouse Station, NJ, USA) are in use in the national immunization programs in Australia, the United States, Latin America, and a few European countries. In these high- and middle-income countries, rotavirus effects have decreased markedly after introduction of the vaccine (*2*–*4*). Universal rotavirus vaccination has not been widely implemented in Asia, and the health effects of rotavirus differ considerably across the continent, with the highest mortality rates concentrated in developing areas. In Central Asia, there are also large variations in the reported rotavirus effects by country (*5*), emphasizing the need for local data to guide the decision on the introduction of the vaccine.

Kazakhstan is the most prosperous country in Central Asia. It has a population of 16 million (*6*) and a land mass equal to approximately half of the continental United States. Kazakhstan has large reservoirs of oil and natural gas and is classified as an upper-middle income economy; its gross national income was US $8,220 per capita in 2011 (*7*), making the country ineligible for international funds to introduce new vaccines. Vaccines included in the national childhood immunization program are fully funded by the government. The health effects of rotavirus in Kazakhstan were estimated at 68 deaths, 4,007 hospitalizations, and 32,500 outpatient visits during 2009 (*5*); another study estimated the total annual cost of rotavirus disease to be US $37.5 million (*8*). No current cost-effectiveness analyses of rotavirus vaccines were available for Kazakhstan.

Recently, 2 economic evaluations of the rotavirus vaccination were conducted in low-income countries in Central Asia (*9*,*10*), but because of differences in rotavirus epidemiology, health care costs, and economy, the results are not generalizable to Kazakhstan. These studies were performed on the basis of static models, which implicitly assume that the probability for disease exposure is constant in time. In contrast, immunization will not only reduce the probability of a vaccinated child to become ill but will also lower the exposure of the virus to others (i.e., herd protection). 

Models that account for changes in transmission over time are referred to as dynamic models. Cost-effectiveness studies of rotavirus vaccination performed on the basis of dynamic transmission modeling were recently used in the United States (*11*), England, and Wales (*12*). To the best of our knowledge, this approach has not been applied in middle-income countries or in settings with a transitional economy. These countries face particular challenges because they are not eligible for international financing of vaccines, and their resources for new health interventions are limited. Rotavirus vaccine effectiveness has been shown to correlate with income level within a country (*13*). It is possible that rotavirus vaccines may perform worse in middle-income settings than in upper-income countries. Hence, scientifically sound estimates of the effect of rotavirus vaccination are in demand.

We present a cost-effectiveness study of rotavirus vaccination in a middle-income country using dynamic modeling. We incorporated direct effects such as death rates and indirect effects such as herd protection of a nationwide vaccination program. Our purpose for the study is twofold: to inform the impending decision on the introduction of rotavirus vaccination into the national immunization program in Kazakhstan, and to compare the cost-effectiveness of a rotavirus vaccination program in a middle-income country with that reported for high-income settings.

## Materials and Methods

We adapted our previously published dynamic model for rotavirus (*14*,*15*) to Kazakhstan. The model is presented in the Technical Appendix.

### Vaccination Parameters

We modeled the effect of introducing the 2-dose rotavirus vaccine Rotarix in the childhood immunization program in Kazakhstan. We implemented vaccination in the model assuming that the vaccine was effective from the first dose at 2 months, similar to other modeling studies (*15*). We chose a 2-dose rotavirus vaccine versus a 3-dose product because it may be more feasible in practice to achieve high coverage for a vaccine requiring fewer doses. Rotarix demonstrated 96% efficacy against rotavirus gastroenteritis (RVGE) hospitalizations in clinical trials and 90% field effectiveness against hospital admissions in high-income European countries (*26*). A lower field effectiveness range of 76%–79% was reported from the middle-income countries in Latin America (*26*–*28*). Because of lack of clinical trials of rotavirus vaccines in countries like Kazakhstan that are in transitional economies, it is difficult to predict the vaccine performance in these settings. On the basis of the aforementioned findings and our own assumptions, we applied a vaccine efficacy of 80% (range 72%–86%) against severe RVGE and 58% (range 51%–64%) against mild RVGE. The vaccine efficacies were varied by varying the proportions of RVGE infection, and severe RVGE infection in vaccinated children (Technical Appendix). We did not adjust the vaccine efficacy for specific rotavirus genotypes because the strains circulating in Kazakhstan are globally common (*29*). Pre- and post-licensure data from developing settings indicate that vaccine protection may wane in the second year of life (*30*,*31*). We conservatively assumed vaccine protection to be 1 year, commencing after administration of the last dose at 4 months of age; that is, children were assumed to be fully protected on average until 16 months of age. However, studies from industrialized settings demonstrated high vaccine efficacy through 3 years of life (*32*,*33*). Therefore, we increased duration of vaccine protection to 2 years in a separate scenario analysis.

The vaccination program in our model was hypothetically initiated on January 1, 2012, with a linear buildup of vaccine coverage during the first 6 months. After this period, the vaccination coverage was assumed to be constant at a fixed level. In Kazakhstan, rotavirus vaccine would be administered concomitantly with the diphtheria–tetanus toxoid–pertussis (DTP) vaccine. The reported coverage for 3 doses of DTP in Kazakhstan is 99% (*7*), although a recent study suggests that only 76% of children 12–60 months of age receive all 3 doses of the DTP vaccine without delay (*34*). Considering age restrictions for the administration of rotavirus vaccines, coverage for the rotavirus vaccine may be lower than for other traditional vaccines administered under the World Health Assembly Expanded Programme on Immunization (*35*) because vaccination may not always be on time. We therefore applied 90% coverage in the base case, but varied coverage between 80% and 100% to explore the effect of this parameter on the cost-effectiveness of vaccination. Similarly to other studies, we did not consider an increased risk for intussusception or any other adverse events after rotavirus vaccination (*2*).

### Disease Outcomes

We calculated the numbers of rotavirus-associated deaths, hospitalizations and outpatient visits in children <5 years from the modeled incidence of severe RVGE (*I_s_*). We assumed that all children with severe RVGE require outpatient care or hospital care, and on the basis of local data, we modeled that 80% of children who were hospitalized with acute diarrhea sought medical care before admission (*8*). We calculated the numbers of rotavirus homecare episodes (without health care encounters) from the modeled incidence of mild RVGE (*I_m_*), whereas the number of rotavirus-associated deaths and hospitalizations was calibrated to the 2009 estimates of 68 (95% CI 63–74) deaths and 4,007 (95% CI 3,740–4,274) hospitalizations based on a recent study from Kazakhstan (*8*) (Technical Appendix).

### Model Uncertainty and Scenario Analyses

We considered uncertainty related to natural history parameters (Table 1), model calibration, and vaccine efficacy and uptake. The role of adults in rotavirus transmission is a key uncertain factor (*15*). Because no sentinel data in this age group were available, we varied the infectiousness of later rotavirus infections relative to that of the primary infection between 1/5 and 1/10 (*14*,*15*). We also varied the mean duration of complete immunity after rotavirus infection from 6 to 12 months. All models were scored according to how well they fit with the sentinel data, adopting a likelihood-based approach by using the Akaike information criterion. In total, 5 candidate models (Technical Appendix Table 1) had support and were simulated, both with and without vaccination. For each model, we calculated the yearly numbers of avoided health outcomes resulting from incidence difference with and without vaccination implemented (Technical Appendix Table 2).

**Table 1:** Natural history and vaccine-related parameters used in dynamic modeling of cost-effectiveness of rotavirus vaccination, Kazakhstan

Parameter	Base value [range]	Reference/source
Demographic		
Population during 1980	15,926 million	(*16*)
Birth cohort*	[217,580–367,750]	(*16,17*)
Mortality rate in <1 y*	20–54 per 1,000 births	(*16,17*)
Mortality rate in 1–4 y*	3.9–6.3 per 1,000 births	(*16,17*)
Net yearly migration rate*	−18.6–0.1 per 1,000	(*16,17*)
Deaths per year*	[128,570–180,000]	(*16,17*)
Natural history		
Duration of maternal protection	70 d	(*18*)
Duration of latency period	0.5 d	(*19*)
Infectious period (days)	8 (first); 6 (second); 4 (later)	(*20*–*22*)
Relative susceptibility	1 (first); 0.62 (second); 0.40 (later)	(*23*)
Relative infectiousness	1 (first); 0.5 (second); [0.1–0.2] (later)	Author assumption
Proportion of infections with RVGE	0.47(first); 0.25 (second); 0.24 (later)	(*23*)
Severe RVGE	0.13 (first); 0.04 (second); 0 (later)	(*23*)
Duration of complete immunity	[6–12 mo]	(*24*)
Vaccination		
Sero-conversion rate	0.96	(*25*)
Relative infectiousness† Relative susceptibility†	0.5 0.62	Assumption Author assumption
Prop. of infections with RVGE	0.30 [0.25–0.35]	(*25*–*27*)
Severe RVGE	0.1175 [0.0885–0.139]	(*25*–*27*)
Coverage	0.9 [0.8–1.0]	Author assumption
Duration of complete immunity	12–24 mo]	Author assumption
Fitted‡		
Infectivity parameter, β0	1.889–2.605	Author calculation
Seasonal forcing, β1	0.025–0.046	Author calculation
Phase angle, θ	0.011–0.251	Author calculation
Mixing (relative susceptibility)§ 0–7m, 8–23 m, 24–35 m	1.077–2.765	Author calculation
*Demographic parameters vary over the time period 1980-2031; only minimum and maximum values are listed in the table. All simulations are performed using the same set of demographic parameters. †Vaccine efficacy calculated for children with no previous natural infection. ‡Details on the fitted parameters of the five candidate models; see corresponding model fits in Technical Appendix Table; seasonal forcing: β0(1 + β1)sin(2πt / 365 + 0). §Relative susceptibility in children <3 years (Technical Appendix, section 1).		

In the economic analysis, we took a weighted average of the incidence differences using Akaike weights (Technical Appendix). We modeled several different scenarios to account for uncertainty in the calibration process, in vaccine efficacy and vaccine uptake (Table 2). In each scenario, we calculated a weighted model as described above. We analyzed a base case (most likely), best-case, and worst-case scenario to account for uncertainty instead of adopting a probabilistic approach because data on vaccine efficacy and rotavirus-associated health outcomes in Kazakhstan are lacking or sparse. In the base case, we used mean estimates for both vaccine efficacy and calibration values. The best-case scenario was based on the highest vaccine efficacy (86% against severe RVGE and 64% against mild RVGE), in combination with the upper bounds of estimated health outcomes (more events to prevent). The worst-case scenario incorporated the lowest vaccine efficacy (72% against severe RVGE and 51% against mild RVGE) and lower estimates of health outcomes (fewer events to prevent). Vaccine coverage was set to 90% in the base case. Scenarios A and B were constructed as described above, by using coverage of 80% and 100%, respectively (Technical Appendix Tables 3, 4). Finally, in Scenario C we extended the vaccine protection period to 2 years in line with data from industrialized settings demonstrating high vaccine efficacy through 3 years of life (*32*,*33*). To estimate indirect or herd protection, we compared predictions of the dynamic model with those of a static cohort model, as was previously suggested (*8*) (Technical Appendix).

**Table 2:** Description of scenarios for the economic evaluation of rotavirus vaccination, Kazakhstan

Scenario	Vaccine parameters					Children <5 y of age, calibration to 2009 sentinel data				
	Mean duration of protection, mo	Coverage	Efficacy against severe RVGE	Efficacy against mild RVGE		Deaths	Hospital admissions	Outpatient clinic visits*	Homecare episodes*	
Base case	12	0.9	0.80	0.58		68	4,007	*I_s_*-0.2*I_h_*	*I_m_*	
Base case, low	12	0.9	0.74	0.51		63	3,740	0.6	0.5*I_m_*	
Base case, high	12	0.9	0.86	0.64		74	4,274	1.4	1.5*I_m_*	
Scenario A	12	0.89	0.80	0.58		68	4,007	*I_s_*-0.2*I_h_*	*I_m_*	
Scenario A, low	12	0.89	0.74	0.51		63	3,740	0.6	0.5*I_m_*	
Scenario A, high	12	0.89	0.86	0.64		74	4,274	1.4(	1.5*I_m_*	
Scenario B	12	1.0	0.80	0.58		68	4,007	*I_s_-*0.2*I_h_*	*I_m_*	
Scenario B, low	12	1.0	0.74	0.51		63	3,740	0.6	0.5*I_m_*	
Scenario B, high	12	1.0	0.86	0.64		74	4,274	1.4	1.5*I_m_*	
Scenario C	24	0.9	0.80	0.58		68	4,007	*I_s_*-0.2*I_h_*	*I_m_*	
Scenario C, low	24	0.9	0.74	0.51		63	3,740	0.6	0.5*I_m_*	
Scenario C, high	24	0.9	0.86	0.64		74	4,274	1.4	1.5*I_m_*	

*RVGE, rotavirus gastroenteritis; *I*_s_, modeled incidence of severe RVGE; *I_h_*, modeled incidence of hospital admissions; *I_m_*, modeled incidence of mild RVGE. †The numbers of outpatient clinic and homecare visits were not calibrated.

### Economic Parameters and Cost-effectiveness Analysis

Direct and indirect costs associated with rotavirus disease were recently estimated in a cost-of-illness study of RVGE in Kazakhstan (*8*). These costs included direct health care and non–health care costs and indirect costs associated with productivity losses due to the work absenteeism of caregivers and rotavirus-related deaths. For this analysis, cost estimates in 2009 US dollars were inflated to 2012 values by using the consumer price index (Table 3). In the absence of a market price for the rotavirus vaccine in Kazakhstan, we used the 2010 price of pneumococcal vaccine (US $43.00 per dose) purchased by the government. Because the vaccine price is a key determinant of cost-effectiveness, we performed various sensitivity analyses with the price ranging from US $1.00 (assuming program price for traditional Expanded Programme on Immunization vaccines) to US $60.00 per dose (considering a price of pneumococcal vaccine that was the most recent vaccine introduced in the program in Kazakhstan). The program costs included the costs of vaccine doses needed to vaccinate the yearly birth cohorts with 2 doses, a 10% vaccine wastage, and an additional US $267,300 to cover the costs of upgrading the cold chain for rotavirus vaccine in the first year of introduction. A 10% loss from vaccine waste was based on published estimates (*36**,**37*). The yearly costs of maintaining the cold chain and the costs of training health personnel were estimated in consultation with the Kazakh Ministry of Health. We assumed that rotavirus vaccination does not incur additional costs to parents because it will be administered concomitantly with other vaccines included in the national immunization program.

**Table 3:** Estimates of projected direct and indirect costs associated with rotavirus disease and rotavirus vaccination, *Kazakhstan*

Item (per case)	Cost estimates in 2012 US dollars			
	Direct	Indirect	Total	Reference/source
Rotavirus death	543.33	67,254.13	67,799.46	(8)
Severe case (inpatient care)	364.36	181.47	545.83	(8)
Moderate case (outpatient care)	32.43	65.57	98.00	(8)
Mild case (homecare)	3.49	21.86	25.35	(8)
Cost of vaccine per dose, base case	43.00	0	43.00	Authors’ assumption
Cold chain upgrade (total first year)	237,300	0	237,300	KMoH
Training costs (first year)	120,096	0	120,096	KMoH
Annual cost of cold chain and training	22,037.74	0	22,037.74	KMoH
*KMoH, Kazakh Ministry of Health.				

We assessed the long-term effect of rotavirus vaccination over a 20-year horizon. All costs and health outcomes were discounted at a rate of 3.0% per year. We conducted cost-effectiveness analyses from the health care system’s perspective (including only direct medical costs) and the societal perspective (including indirect costs) using life-years gained as a measure of benefit. We estimated a break-even price for the rotavirus vaccine, in which the total health care costs of the vaccination program were equal to the expected cost savings for the health care system. All results are expressed as mean values with a range to represent realistic vaccination outcomes given the uncertainty in the epidemiologic model. Lacking actual data on uncertainty in the parameter values, we could not express uncertainty in terms of statistical distributions, so we chose to use 1-way sensitivity analyses.

## Results

### Base Case

**Figure 1:**
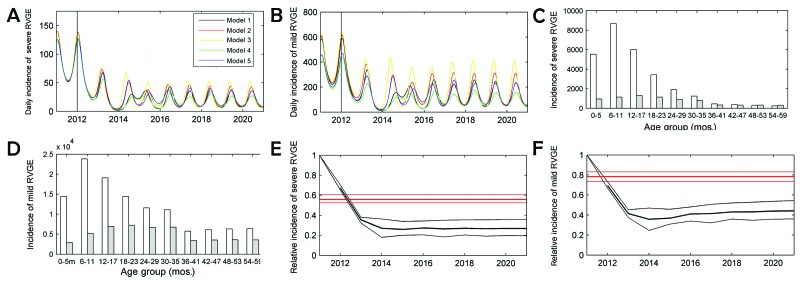
Projected epidemiologic effect of rotavirus vaccination in children <5 years of age in Kazakhstan. A) Estimated daily incidence of severe RVGE (base case scenario) with introduction of rotavirus vaccination in January 2012 in the 5 candidate models. B) Estimated daily incidence of mild RVGE (base case) with introduction of the rotavirus vaccination in January 2012 in the 5 candidate models. C) Yearly age-specific incidence of severe RVGE pre-vaccination (white) and 10 years postvaccination (gray). D) Yearly age-specific incidence of mild RVGE pre-vaccination (white) and 10 years postvaccination (gray). E) Relative incidence of severe RVGE with vaccination compared with the expected incidence without vaccination; the blue curve shows the mean relative incidence with lower and upper bounds predicted by the synthesis of dynamic models, including both direct and indirect effects, while the red curve shows the relative incidence predicted by a static cohort model incorporating only the direct effects (Technical Appendix). F) Relative incidence of mild RVGE with vaccination compared with the expected incidence without vaccination; the blue curve shows the mean relative incidence with lower and upper bounds in the synthesis of dynamic models; the red curve shows the relative incidence predicted by a static cohort model.

Our model projects that the introduction in Kazakhstan of routine rotavirus immunization with 90% coverage and a mean duration of vaccine-induced protection of 1 year would reduce the incidence of severe and mild RVGE in children <5 years of age within the first year (Figure 1, panels A, B). After ≈4 years of administration of the vaccine program, the dynamics of rotavirus would stabilize, and infection would occur with yearly oscillations. Before the start of vaccination, the peak incidence of RVGE would be among children <12 months of age. The highest incidence of severe and mild disease postvaccination is found during the second and third years of life, respectively (Figure 1, panels C, D). The age shift is predicted to occur within 3 years of vaccination. The yearly peak is predicted to be delayed by 14–20 weeks compared with the epidemic peak timing without vaccination (Technical Appendix Figure 4). 

During 20 years of vaccination, the predicted incidence of severe RVGE would be reduced by 74% (base case range 64%–80%) of prevaccine levels. The incidence of mild RVGE would be reduced by 56% (range 45%–64%) compared with incidence among unvaccinated children (Figure 1, panels E, F). Our model predicts substantial indirect or herd protection conferred by rotavirus vaccination. The indirect effects account for ≈40% (range 25–33% in relative terms) of the reduction in the projected incidence of severe RVGE, whereas 60% (range 0.28–0.38 in relative terms) of the incidence drop in mild RVGE would be caused by a reduced circulation of rotavirus. Our model projects that over 20 years, a vaccination program with 90% coverage would prevent 881 (range 776–1,004) deaths, 51,891 (range 46,094–57,971) hospital admissions, 370,268 (range 211,825–541,919) outpatient clinic visits, and 1.345 (range 0.641–2.112) million homecare episodes. These values correspond to ≈74% (range 70%–77%) averted deaths, hospitalizations, and outpatient clinic visits and 55% (range 53%–58%) averted homecare episodes compared with the values predicted without vaccination. In that time period, 54,784 (range 48,304–62,442) undiscounted life-years are saved (Table 4).

**Table 4:** Estimated projected costs and avoided health outcomes of rotavirus vaccination program in Kazakhstan, 2012–2031

Outcome	No vaccination	Base case, 90% vaccine coverage, 1-y vaccination protection				Scenario A, 80% vaccine coverage, 1-y vaccination protection				Scenario B, 100% vaccine coverage, 1-y vaccination protection				
		Mean	Low	High		Mean	Low	High		Mean		Low		High
		Avoided outcomes, undiscounted												
Fatal cases	1,310	880	776	1,004		777	681	890		985	876		1 114	
In-hospital care	77,205	51,891	46,094	57,971		45,802	40,436	51,447		58,086	52,038		64,396	
Out-patient visits	550,896	370,268	211,825	541,919		326,820	185,825	480,935		414,473	239,145		601,983	
Home care episodes	2,675,456	1,344,747	640,836	2,112,400		1,163,780	552,160	1,835,487		1,544,096	740,039		2,412,843	
Life years gained		54,784	48,304	62,442		48,356	42,375	55,416		61,325	54,534		69,363	
Vaccination		530.7	530.7	530.7		471.9	471.9	471.9		589.6	589.6		589.6	
In-hospital care	−25.7	−18.9	−16.8	−21.2		−16.7	−14.7	−18.7		−21.2	−18.9		−23.5	
Out-hospital care	−16.3	−12.0	−6.9	−17.6		−10.6	−6.0	−15.6		−13.4	−7.8		−19.5	
Homecare	−8.5	−4.7	−2.2	−7.4		−4.1	−1.9	−6.4		−5.4	−2.6		−8.4	
Indirect costs	−179.4	−122.3	−88.4	−159.7		−107.4	−77.4	−140.9		−137.7	−100.3		−178.9	
Total net costs	229.9	372.8	416.4	325.0		333.1	371.8	290.2		411.9	460.1		359.3	
	Incremental cost-effectiveness ratios, societal perspective													
Discounted 3%	NA	18,044	22,779	13,854		18,280	27,991	13,955		17,775	22,250		13,768	
	Incremental cost-effectiveness ratios, health care perspective													
Discounted 3%	NA	23,892	27,573	20,557		24,102	23,210	20,620		23,658	27,061		20,526	
	Threshold prices, 3% discounting													
Medical break-even price†	NA	$2.78	$2.01	$3.62		$2.78	$1.96	$3.60		$2.83	$2.05		$3.65	

*Negative values indicate prevented or avoided costs. NA, not applicable. †The price per vaccine dose at which the vaccinations costs are offset by cost saving generated from lower morbidity and mortality rates.

**Figure 2:**
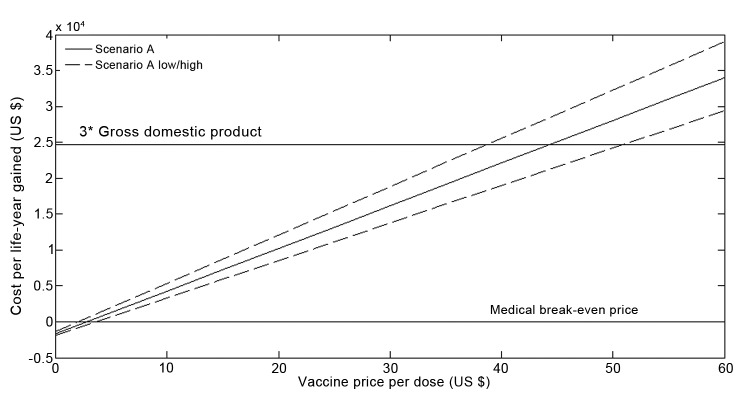
Projected cost (US $) per life-year gained over a 20-year time period (2012–2031) after introduction of rotavirus vaccination in Kazakhstan, according to purchasing price of 1 vaccine dose.

In the base case, the net undiscounted program costs would be US $530.7 million; the net costs when accounting for cost savings would be US $372.8 (range $325.0–$416.4) million (Table 4). These results would imply a cost of US $23,892 per life-year saved (i.e., incremental cost-effectiveness ratio of US $23,892) in a health care perspective (range $20,557–$27,573) and US $18,044 in a societal perspective ($13,854–$22,779); both estimates were discounted at 3%. Figure 2 shows the cost per life-year gained as a function of the vaccine price per dose in a 20-year perspective. At a cost of US $2.78 (range $2.01–$3.62), the additional cost of the vaccination program would be entirely offset by the cost savings to the health care system corresponding to the medical break-even price.

### Scenario Analyses

Varying the vaccination coverage between 80% and 100% (scenarios A and B) did not substantially influence the cost-effectiveness ratio (Table 4). For example, increasing the coverage to 100% generated only a moderate decrease in the cost per life-year gained (US $23,658 in a health care perspective and US $17,775 in a societal perspective); a marginal change in the cost-effectiveness ratio was also observed when vaccine coverage was decreased to 80%. We included 6 years without vaccination to explore any carryover effects after vaccination is discontinued. The model predicts that such effects are small because infection rates return to prevaccine levels quickly.

Lastly, in scenario C we simulated a 90% vaccine coverage assuming 2-year mean vaccine protection (Table 5; Technical Appendix Table 5; Technical Appendix Figure 3). These results suggest a cost of US $22,579 per life-year saved in a health care perspective and US $16,775 in a societal perspective; the medical break-even price was estimated at a cost of US $ 2.95 per dose. Compared with the base case (Table 4), assuming 2 years of vaccination protection reduced the cost per life-year saved by 5.5% in a health care perspective and 7.4% in a societal perspective.

**Table 5:** Estimated projected costs in US dollars and avoided health outcomes from rotavirus vaccination with 2-year protection, Kazakhstan, 2012–2031*

Outcome	No vaccination	Scenario C, 90% vaccine coverage, 2-y vaccination protection		
		Mean	Low	High
		Avoided outcomes, undiscounted		
Deaths	1,310	919	823	1,034
Hospital admissions	77,205	54,163	48,823	59,701
Out-patient visits	550,896	386,479	224,410	558,096
Home care episodes	2,675,456	1,544,202	747,494	2,390,646
Life-years gained	NA	57,183	51,174	64,306
		Avoided costs, undiscounted, US $43 per vaccine dose		
Vaccination	NA	530.7	530.7	530.7
Prevented in-hospital care	25.7	19.7	17.8	21.8
Prevented outpatient care	16.3	12.5	7.3	18.1
Prevented homecare	8.5	5.4	2.6	8.4
Avoided indirect costs	179.4	130.7	95.2	169.2
Total net costs in US$	229.9	362.4	407.8	313.4
		Incremental cost-effectiveness ratios, societal perspective		
Discounted 3%	NA	16,775	21,031	12,952
		Incremental cost-effectiveness ratios, health care perspective		
Discounted 3%	NA	22:759	25:898	19:841
		Threshold prices, 3% discounting		
Medical break-even price†	NA	$2.95	$2.15	$3.79

*NA, not applicable. †The price per vaccine dose at which the vaccinations costs are offset by cost saving generated from lower morbidity and mortality rates.

## Discussion

Our study evaluated the cost-effectiveness of rotavirus vaccination in a middle-income country by use of a dynamic model. The results indicate that universal rotavirus vaccination in Kazakhstan could prevent 800–1,000 deaths, 46,000–58,000 hospitalizations, 210,000–540,000 outpatient clinic visits, and 0.6–2.1 million homecare episodes during the next 2 decades. Our study suggests that the cost-effectiveness of rotavirus vaccination is determined by 2 key factors: the ability of the vaccines to prevent severe RVGE in children and the market price of the vaccine. 

Vaccination also reduces productivity losses because of lower mortality rates and less work absenteeism among parents. However, the small difference between the cost-effectiveness ratios with and without indirect costs is explained by the dominant role of the vaccine costs. In the economic analysis, we calculated the break-even price, representing the price at which the costs of vaccination would be offset by the health care cost savings from avoided cases. With vaccine prices below the break-even price, the vaccination program would become one of cost saving. We believe that estimating cost per life-year gained and the break-even price of vaccine is informative for decision makers negotiating the price with manufacturers in the absence of an established market price for the product. Whether the cost per life-year represents value for money and is considered cost-effective is a political question for Kazakhstan authorities to decide. WHO has suggested that governments should be willing to pay 3 times gross domestic product per capita per year for a good life-year. For Kazakhstan, this would amount to US $24,660.

A strength of this study is that the results are a synthesis of 5 models and use a likelihood-based approach, in which the models are weighted according to their ability to fit the sentinel data. This approach is common in weather and finance models but not in infectious disease modeling.

Our model demonstrates the role of indirect protection conferred by rotavirus vaccination. In our base case scenario, herd protection accounted for a 40% reduction in the incidence of severe RVGE and a 60% decrease in the incidence of mild RVGE. The contribution of indirect effects to the overall effect of vaccine is an observation also reported by other dynamic modeling studies and supported by empirical data from countries already using rotavirus vaccine in routine immunization programs (*2*,*3*). The incidence reduction in our model is larger than that found by Atkins et al. in a study from England and Wales, where 25% and 40% of the incidence reduction in severe and mild RVGE, respectively, were accounted for by herd protection, assuming a 1-year mean vaccine protection (*38*). This difference may be attributed to differences in assumptions on vaccine-related parameters, the magnitude of the disease burden, population dynamics and other model characteristics. This model has previously been fitted to data from England and Wales with a basic reproductive number of the primary infection of *R*_0_ = 17.6 (*15*), which is smaller than the value estimated for Kazakhstan of *R*_0_ = 19.2 − 2104, thereby suggesting higher transmission pressure in the latter setting. 

Data from Finland suggest that vaccine protection may last for >1 year (*39*). We have also tested the model assuming 2 years of vaccine-derived protection. In this case, we found less indirect protection against severe RVGE, roughly representing 20% of the reduction (Table 5; Technical Appendix Figure 3). The direct effect from vaccination increases because it is calculated from the expected infections in vaccinated children 2–28 months of age, had they not been vaccinated, versus children 2–16 months of age in the base case. We obtained a modest effect from extending the vaccine protection period by 1 year, which may be related to our use of a mean value for the vaccine duration instead of a fixed duration of vaccine protection, implying that some children will experience a shorter duration of protection.

We decided to provide a conservative estimate of the cost-effectiveness of rotavirus vaccination. First, we assumed that direct vaccine-derived protection lasts for 1 year because data on a longer duration of vaccine protection from industrialized countries may not be directly generalizable to Kazakhstan. Second, we assumed that the risk for severe RVGE is age-independent. Vaccination increases the average age of infection, and it is plausible that this risk for severe RVGE is lower for older versus younger children. Third, we applied a lower estimate of vaccine efficacy in our model because Kazakhstan is a developing nation. However, if rotavirus vaccines demonstrate a better efficacy in this country, it may substantially influence the cost-effectiveness of vaccination.

Several limitations in our study warrant care in interpretation of the results. First, our model was fitted to the 2-year sentinel hospital data on rotavirus surveillance on the basis of information from 2 hospitals; hence, changes in annual RVGE incidence and seasonality may have not been fully captured. Likewise, local data on outpatient visits are sparse. We have attempted to compensate for this by using wide upper and lower confidence bounds on these estimates. Second, the parameters used to characterize natural rotavirus infections were based on those in a study conducted in Mexico and may not properly represent the epidemiology of rotavirus infections in Central Asia. Third, we used continuous aging in the model, which may have introduced a bias arising from persons aging at different rates. Even so, we used a small age band of 1 month, and we tested the model performance without finding severe bias (*14*). Fourth, the choice of simulation period may imply that carryover effects beyond 20 years were disregarded, but the scenario analysis indicates that such effects are small and will not influence the ICERs because of discounting. Fifth, we tested the uncertainty of the vaccine price in the sensitivity analysis, but because of lack of data, we were unable to test the uncertainty of other cost parameters in the model. Finally, because of lack of data, we disregarded improved quality of life in the economic analysis.

In conclusion, rotavirus vaccination in Kazakhstan will provide considerable direct health benefits in terms of reduced illness and deaths. Using the WHO criterion for cost-effectiveness, vaccination would be considered cost-effective under most of the assumptions of our analyses. With a low vaccine price, the avoided disease costs from vaccination will be greater than the vaccination costs. Further study is warranted to measure the benefits of herd immunity conferred by vaccination and to add that information to the current comparison of the costs of illness to those of a national vaccine program.

Technical Appendix Detailed description of dynamic model for estimating cost-effectiveness of rotavirus vaccination in Kazakhstan.
