# Supplementary material for: Dynamic Modeling of Cost-effectiveness of Rotavirus Vaccination, Kazakhstan
Source: Emerg Infect Dis. 2014 Jan;20(1):29–37. doi: 10.3201/eid2001.130019 (PMC3884708; doi:10.3201/eid2001.130019)
Supplement: Technical Appendix — Detailed description of dynamic model for estimating cost-effectiveness of rotavirus vaccination in Kazakhstan. [file 13-0019-Techapp-s1.pdf]

Article DOI: http://dx.doi.org/10.3201/eid2001.130019

Dynamic Modeling of Cost-effectiveness of Rotavirus Vaccination in Kazakhstan

Technical Appendix

Dynamic Model Description and Usage

1. Transmission Dynamic Model

We adapted our previously published dynamic model for rotavirus (*1*,*2*) to Kazakhstan. Rotavirus infection dynamics is complex because both symptomatic and asymptomatic re-infections occur. The susceptibility to new infections and disease severity tend to decline after the first infection (*3*,*4*). In our model, natural rotavirus infection is grouped into three infection types with distinct epidemiological characteristics: primary, secondary and later infections. All infections following a common Susceptible-Exposed-Infected-Recovered (SEIR) structure (Technical Appendix Figure 1). For each infection type, we distinguish between cases of severe rotavirus gastroenteritis (RVGE), mild RVGE and asymptomatic infection using published data on natural history (*3*). The first infection may cause severe RVGE, mild RVGE, or be asymptomatic. The second infection has similar outcomes, but with a lower probability for severe RVGE. Subsequent infections are assumed to cause age-independent proportions of mild and asymptomatic cases. Children are assumed to be protected against RVGE at birth by maternal antibodies for an average of 70 days; the mean protection duration was estimated based on the average period of exclusive breastfeeding in Kazakhstan (*5*).

We modeled the population using monthly age groups for 0-4 years and 10-yearly age groups for 5-14 years, 15-24 years, 25-34 years and 35-44 years; the remaining population above 45 years of age was modeled as one group. We considered age-specific mortality for children <1 year and 1-4 years of age, whereas deaths in those above five years of age were assumed to occur only in the oldest age group (text Table 1). We fitted

the model to the weekly, age-stratified RVGE hospitalizations in children <5 years of age collected from two sentinel hospitals in Kazakhstan from 2007-2009 (*6,7*).

2. Model Fitting and Model Selection

We fitted the model output of severe rotavirus gastroenteritis (RVGE) in children < 5 years old to hospital sentinel data from Kazakhstan, assuming that the pediatric hospitalizations represented a time-independent proportion p of the modeled severe infections. The sentinel study was conducted during 113 weeks from week 44 in 2007 through week 52 in 2009, and included a total of 1,012 children hospitalized with rotavirus-associated gastroenteritis.

We utilized a maximum likelihood approach, assuming that the hospital admissions $h(a,t)$ in age group a and at time point t were Poisson-distributed, with a mean equal to the corresponding incidence of severe infections $s(a,t;\theta)$ predicted by the model. The likelihood function was given by:

$$L(\theta) = \sum_{t=1}^{113}\sum_{a=1}^{60} h(a,t)\log s(a,t;\theta) - s(a,t;\theta) - \sum_{j=1}^{s(a,t;\,\theta)} \log j \quad (1.1)$$

The parameters to be fitted, $\theta = \{p, \beta 0, \beta 1, \varphi, a_{0-7}, a_{8-23}, a_{24-35}\}$, included the proportion of severe RVGE cases represented in the sentinel hospitalization data (p), an infectivity parameter $(\beta 0)$ and seasonal forcing $\beta 1$ and phase φ described by:

$$\beta 0(1+\beta 1(\sin(2\pi t/365+\varphi))) \quad (1.2)$$

We assumed that children <3 years of age had a higher risk of acquiring infection because rotavirus has a fecal-oral transmission route, and young children have a tendency to put fingers and objects in the mouth. This effect was implemented by fitting parameters for higher transmission to 0-7-month-old children, 8-23-month-old children and 2-year-old children $(a_{0-7}, a_{8-23}, a_{24-35})$. In the preliminary analyses we also tested other assumptions for mixing in the population, including homogeneous mixing and assortative mixing. However, we obtained the best fit by incorporating elevated

transmission to the youngest age groups, and we employed this model structure to make predictions.

To account for uncertainty in the duration of complete immunity following infection, we fitted the model by varying this time period from 6 to 12 months, and we varied the infectiousness in “later” infections relative to that of the primary infection between 1/5 – 1/10. All models were scored using the Akaike Information Criterion (Technical Appendix Table 1):

$$AIC = 2k - 2\ln L(\hat{\theta}) \quad (1.3)$$

where *k* is the number of fitted model parameters and $L(\hat{\theta})$ is the maximized likelihood.

Five of the candidate models had support $(AIC_j - \min AIC < 5)$, and they were all used to make predictions according to their Akaike weights:

$$w_i = \frac{\exp(-\Delta_i / 2)}{\sum_j \exp(-\Delta_j / 2)}; \qquad \Delta_i = AIC_i - \min AIC \quad (1.4)$$

The fitted models reproduced well the age distribution of pediatric hospitalizations in the sentinel data (Technical Appendix Figure 2, panel A), although with a slight tendency to both over- and underestimate cases in children < 1 year, and 1-year old children, respectively. The models predicted a seasonal peak in late January (week 4). The bi-modal seasonal pattern indicated in the sentinel data (Technical Appendix Figure 2, panel B), which may relate to climatic differences between the central and south region of Kazakhstan where the sentinel hospitals are situated, were not reproduced in the models because seasonal forcing was incorporated using a simple sinusoidal forcing function.

3. Model Calibration

We calibrated the number of deaths and hospital admissions from the incidence of severe RVGE in the model $s(a,t)$ with the use of published estimates for Kazakhstan in 2009 of 68 (63-74) deaths and 4007 (3740-4272) hospital admissions attributable to rotavirus in children < 5 years old. We assumed that age- and time-independent proportions p_d, p_h of

young children with severe RVGE die and become admitted to a hospital, respectively. All children <5 years of age with severe RVGE were assumed to seek outpatient medical care or be hospitalized or both, and based on local data, we assumed that 80% of hospitalized children sought medical care prior to admission (*8*). Lastly, we estimated the number of homecare episodes from the predicted incidence of mild infections $m(a,t)$:

$$deaths(a,t) = p_d s(a,t); \quad p_d = \frac{68}{\sum_{2009}\sum_{<5y} s(a,t)}; \quad low\ /\ high\,(63\ /\ 74)$$

$$hosp(a,t) = p_h s(a,t); \quad p_h = \frac{4007}{\sum_{2009}\sum_{<5y} s(a,t)}; \quad low\ /\ high\,(3764\ /\ 4267)$$

$$outp(a,t) = s(a,t) - (1-0.8)\ hosp(a,t); \quad low\ /\ high\,(-\ /\ +40\%)$$

$$home(a,t) = m(a,t); \quad low\ /\ high\,(-\ /\ +50\%) \qquad (1.5)$$

Because outpatient clinic visits and homecare episodes were not calibrated, we used their values to validate the models. In the reference year 2009, the models predicted between 28,592-30,189 outpatient clinic visits and 105,850-133,074 homecare episodes using the mean calibration values. The values are consistent with published estimates for Kazakhstan in that year of 32,500 (18,700-42,700) outpatient clinic visits and 130,000 (56,100-213,700) homecare episodes (*9*).

For all five candidate models $j = 1..5$, and

all scenarios *S*, we annually subtracted the number of predicted health outcome events $E_{Sj}^{V}(y)$, with vaccination implemented from the number of events predicted by the model without intervention $E_{Sj}^{0}(y)$. The estimation of yearly avoided health outcomes was calculated as follows:

$$\Delta E_S^V(y) = \sum_j w_j \left(E_{Sj}^0(y) - E_{Sj}^V(y)\right) \qquad (1.6)$$

where w_j represents the Akaike weights for the candidate models and y is the years from 2012-2031. Tables 2–4 present the predicted incremental number of avoided health

outcomes from 2012-2031 in the base case (Scenario A) and the sensitivity analyses (Scenario B-C).

4. Direct Effects

We used the method suggested by Atkins et al. (*10,11*) to estimate the direct effects of vaccination. In this approach, the direct effects are estimated from the long-term annual number of vaccinated children who would have been infected had vaccination not been implemented. This number is calculated as follows:

$$\Delta I_X^{direct} = \sum_{a=2m}^{16m} \left(\phi VE_X \sum_{j'} \delta I_X^{novacc}(j') \right) \quad (1.7)$$

where ϕ is the vaccine coverage, $VE_X \quad X = \{mild, sev\}$ is the vaccine efficacy and δI_X^{novacc} denotes the incidence in the absence of vaccination at day j'. The expression is summed over all age groups between 2 to 16 months of age, assuming that vaccination is effective from the first dose and that vaccination protects against infection for a mean duration of 12 months following the last vaccine dose scheduled at 4 months of age. This way of calculating the direct effects is identical to the vaccine effect predicted by a static cohort model (*10,11*).

5. Vaccine efficacy

Vaccine efficacy against any infection was calculated as:

$$sero(1-sus_v) = 0.96(1-0.62) = 0.3648 \quad (1.8)$$

where $sero$ is the sero-conversion rate and sus_v is the relative susceptibility against infection in vaccinated children (Technical Appendix Table 1).

Vaccine efficacy against RVGE infection was calculated as:

$$\begin{aligned} &sero(1-sus_v(prop_{RVGE\_V} / prop_{RVGE\_1})) = \\ &0.96(1-0.62(0.30/0.47)) = 0.58 \end{aligned} \quad (1.9)$$

here $prop_{RVGE\_V}$ is the proportion of RVGE infections experienced in vaccinated children during their first infection following immunization and $prop_{RVGE\_1}$ is the proportion of RVGE infections in primary natural infection. The numbers correspond to the base case assumption (Technical Appendix Table 1).

Vaccine efficacy against severe RVGE infection was calculated as:

$$sero(1 - sus_v((prop_{sev\_V}\,prop_{RVGE\_V})/(prop_{sev\_1}\,prop_{RVGE\_1}))) =$$
$$0.96(1 - 0.62((0.1175 * 0.30)/(0.28 * 0.47))) = 0.80 \qquad (1.10)$$

where $prop_{sev\_V}$ is the proportion of severe RVGE infections in vaccinated children during their first infection following immunization, and $prop_{sev\_1}$ is the proportion of severe RVGE infections during primary natural infection. The numbers correspond to base case assumption (Technical Appendix Table 1).

References

1. de Blasio BF, Kasymbekova K, Flem E. Dynamic model of rotavirus transmission and the impact of rotavirus vaccination in Kyrgyzstan. Vaccine. 2010;28:7923–32. PubMed http://dx.doi.org/10.1016/j.vaccine.2010.09.070

2. Pitzer VE, Atkins KE, de Blasio BF, Van ET, Atchison CJ, Harris JP, et al. Direct and indirect effects of rotavirus vaccination: comparing predictions from transmission dynamic models. PLoS ONE. 2012;7:e42320. PubMed http://dx.doi.org/10.1371/journal.pone.0042320

3. Velázquez FR, Matson DO, Calva JJ, Guerrero L, Morrow AL, Carter-Campbell S, et al. Rotavirus infections in infants as protection against subsequent infections. N Engl J Med. 1996;335:1022–8. PubMed http://dx.doi.org/10.1056/NEJM199610033351404

4. Velazquez FR. Protective effects of natural rotavirus infection. Pediatr Infect Dis J. 2009;28:S54–6. PubMed http://dx.doi.org/10.1097/INF.0b013e3181967c03

5. United Nations Children's Fund (UNICEF), Agency of the Republic of Kazakhstan on Statistic. Kazakhstan Multiple Indicator Cluster Survey 2006. 2007. [cited 2012 Dec 05] http://www.childinfo.org/files/MICS3_Kazakhstan_FinalReport_2006_Eng.pdf

6. Latipov R, Utegenova E, Kuatbayeva A, Kasymbekova K, Abdykarimov S, Juraev R, et al. Epidemiology and burden of rotavirus disease in Central Asia. Int J Infect Dis. 2011;15:e464–9. PubMed http://dx.doi.org/10.1016/j.ijid.2011.03.014

7. Latipov R, Kuatbaeva A, Kristiansen O, Aubakirova S, Akhanaeva U, Kristiansen IS, et al. Economic burden of rotavirus disease in children under 5 years in Kazakhstan. Vaccine. 2011;29:4175–80. PubMed http://dx.doi.org/10.1016/j.vaccine.2011.03.019

8. Latipov R, Kuatbaeva A, Kristiansen O, Aubakirova S, Akhanaeva U, Kristiansen IS, et al. Economic burden of rotavirus disease in children under 5 years in Kazakhstan. Vaccine. 2011;29:4175–80. PubMed http://dx.doi.org/10.1016/j.vaccine.2011.03.019

9. Latipov R, Utegenova E, Kuatbayeva A, Kasymbekova K, Abdykarimov S, Juraev R, et al. Epidemiology and burden of rotavirus disease in Central Asia. Int J Infect Dis. 2011;15:e464–9. PubMed http://dx.doi.org/10.1016/j.ijid.2011.03.014

10. Atkins KE, Shim E, Carroll S, Quilici S, Galvani AP. The cost-effectiveness of pentavalent rotavirus vaccination in England and Wales. Vaccine. 2012. PubMed

11. Atkins KE, Shim E, Pitzer VE, Galvani AP. Impact of rotavirus vaccination on epidemiological dynamics in England and Wales. Vaccine. 2012;30:552–64. PubMed http://dx.doi.org/10.1016/j.vaccine.2011.11.064

Technical Appendix Table 1. Fitted model parameters and model fit

Model			Transmission			Relative susceptibility			Fit	
j	inf_{later}	*duration imm.*	β0	β1	φ	a_0–11m	a_12–23m	a_24–35m	AIC	Weight (w)
1	1/10	6m	2.60514	0.03399	0.04981	1.28758	2.43732	2.11278	3854.28750	0.3683
2	1/7	6m	2.55604	0.03124	0.12781	1.07736	2.06568	1.88822	3854.39829	0.3480
3	1/5	6m	2.12452	0.04565	0.25094	1.10342	2.13370	1.93065	3855.00434	0.2574
4	1/7	12m	1.99394	0.02614	0.00034	2.02571	3.97314	3.22687	3860.86022	0.0138
5	1/5	12m	1.88881	0.02483	0.01098	1.47222	2.99835	2.76524	3861.10907	0.0122

Technical Appendix Table 2. Predicted incremental avoided health outcomes from 2012–2031 for base case scenario with 90% vaccination coverage and a mean duration of 1 year of complete vaccine protection

Year	Cumulative avoided deaths			Cumulative avoided hospitalizations			Cumulative avoided outpatient visits			Cumulative avoided homecare episode.		
	Base case	Low	High	Base case	Low	High	Base case	Low	High	Base case	Low	High
2012	23	22	26	1 378	1 282	1 475	9,835	5 893	13 787	41 375	20 633	62 227
2013	68	62	75	3 999	3 693	4 309	28,532	16 972	40 279	121 303	60 195	183 332
2014	120	110	133	7 092	6 507	7 690	50,602	29 903	71 884	208 827	102 882	317 868
2015	170	154	189	10 023	9 135	10 938	71,520	41 978	102 253	292 966	143 497	448 471
2016	218	196	244	12 861	11 648	14 117	91,769	53 527	131 970	370 429	180 415	570 145
2017	267	239	300	15 749	14 213	17 345	112 380	65 314	162 145	446 319	216 488	689 636
2018	314	280	354	18 496	16 630	20 439	131 980	76 424	191 065	518 702	250 770	803 977
2019	360	321	407	21 235	19 051	23 513	151 526	87 550	219 800	589 081	284 091	915 189
2020	405	360	459	23 882	21 385	26 488	170 407	98 275	247 610	656 973	316 210	1 022 548
2021	449	399	509	26 465	23 664	29 392	188 844	108 750	274 758	722 664	347 269	1 126 479
2022	492	437	558	29 013	25 914	32 252	207 022	119 090	301 501	786 791	377 589	1 227 939
2023	535	474	607	31 504	28 113	35 052	224 797	129 192	327 668	849 096	407 028	1 326 574
2024	577	511	655	33 990	30 309	37 842	242 534	139 283	353 752	910 631	436 106	1 423 980
2025	620	548	704	36 507	32 533	40 668	260 498	149 505	380 172	972 596	465 398	1 522 036
2026	663	586	754	39 071	34 797	43 547	278 794	159 911	407 087	1 035 200	494 982	1 621 131
2027	707	624	804	41 641	37 064	46 435	297 128	170 328	434 085	1 097 525	524 395	1 719 905
2028	750	662	854	44 198	39 318	49 312	315 375	180 685	460 975	1 159 417	553 571	1 818 091
2029	794	700	904	46 761	41 577	52 196	333 666	191 066	487 936	1 221 385	582 769	1 916 435
2030	837	738	954	49 327	43 836	55 084	351 972	201 450	514 931	1 283 226	611 889	2 014 639
2031	881	776	1 004	51 891	46 094	57 971	370 268	211 825	541 919	1 344 748	640 836	2 112 400

Technical Appendix Table 3. Predicted incremental avoided health outcomes from 2012–2031 for scenario A with 80% vaccination coverage and a mean duration of 1 y of complete vaccine protection

Year	Cumulative avoided deaths			Cumulative avoided hospitalizations			Cumulative avoided outpatient visits			Cumulative avoided homecare episodes		
	A	Low	High	A	Low	High	A	Low	High	A	Low	High
2012	21	20	23	1 260	1 172	1 349	8 992	5 386	12 611	37 525	18 707	56 456
2013	62	57	68	3 647	3 366	3 934	26 026	15 468	36 773	110 791	54 952	167 527
2014	104	94	115	6 100	5 557	6 660	43 529	25 538	62 258	181 271	89 048	276 704
2015	149	133	166	8 753	7 923	9 613	62 456	36 408	89 862	253 166	123 520	389 002
2016	192	171	216	11 304	10 175	12 479	80 661	46 758	116 656	321 512	156 018	496 581
2017	234	208	264	13 764	12 327	15 263	98 213	56 650	142 684	386 269	186 605	599 128
2018	275	244	312	16 220	14 481	18 037	115 735	66 550	168 613	448 830	216 101	698 362
2019	316	280	360	18 639	16 605	20 768	132 997	76 310	194 139	509 634	244 754	794 852
2020	356	314	405	20 972	18 650	23 406	149 648	85 707	218 804	568 155	272 300	887 813
2021	395	348	450	23 256	20 653	25 987	165 946	94 911	242 932	624 857	298 978	977 920
2022	433	381	494	25 511	22 632	28 532	182 034	104 007	266 724	680 234	325 032	1 065 925
2023	470	414	537	27 719	24 570	31 026	197 792	112 913	290 033	734 079	350 350	1 151 537
2024	508	446	580	29 919	26 502	33 508	213 488	121 791	313 239	787 315	375 388	1 236 169
2025	546	480	624	32 157	28 469	36 031	229 453	130 829	336 823	841 092	400 697	1 321 605
2026	584	513	668	34 435	30 471	38 601	245 710	140 029	360 845	895 513	426 306	1 408 072
2027	623	547	713	36 714	32 471	41 174	261 972	149 220	384 899	949 602	451 723	1 494 124
2028	662	580	757	38 981	34 459	43 736	278 150	158 357	408 849	1 003 232	476 893	1 579 540
2029	700	614	802	41 254	36 452	46 305	294 371	167 516	432 865	1 056 932	502 085	1 665 097
2030	739	648	846	43 530	38 446	48 877	310 605	176 678	456 910	1 110 513	527 206	1 750 513
2031	777	681	891	45 802	40 436	51 447	326 820	185 826	480 936	1 163 780	552 160	1 835 487

Technical Appendix Table 4. Predicted incremental avoided health outcomes from 2012–2031 for scenario B with 100% vaccination coverage and a mean duration of 1 y of complete vaccine protection

Year	Cumulative avoided deaths			Cumulative avoided hospitalizations			Cumulative avoided outpatient visits			Cumulative avoided homecare episodes		
	B	low	high	B	low	high	B	low	high	B	low	high
2012	25	23	27	1 483	1 380	1 587	10 584	6 344	14 831	44 897	22 396	67 506
2013	74	68	82	4 375	4 046	4 708	31 216	18 594	44 011	132 145	65 615	199 599
2014	138	127	151	8 135	7 544	8 732	58 049	34 669	81 631	245 562	121 722	371 542
2015	194	177	214	11 437	10 534	12 358	81 611	48 407	115 525	335 603	165 107	511 547
2016	249	226	276	14 655	13 422	15 918	104 571	61 683	148 808	430 422	210 951	658 502
2017	304	275	338	17 900	16 339	19 505	127 723	75 086	182 333	514 074	250 707	790 236
2018	355	321	397	20 941	19 028	22 916	149 425	87 446	214 223	599 331	291 471	923 755
2019	408	367	456	24 043	21 808	26 355	171 561	100 220	246 371	678 732	329 188	1 048 845
2020	457	410	512	26 931	24 357	29 600	192 169	111 933	276 704	757 566	366 769	1 172 645
2021	507	455	569	29 871	26 989	32 861	213 148	124 030	307 191	832 037	402 128	1 290 021
2022	554	496	623	32 639	29 432	35 970	232 893	135 256	336 249	906 334	437 519	1 406 776
2023	602	538	677	35 469	31 966	39 110	253 089	146 901	365 602	976 914	471 005	1 518 094
2024	648	579	730	38 169	34 350	42 141	272 355	157 858	393 945	1 048 124	504 904	1 630 064
2025	696	622	784	41 017	36 900	45 301	292 677	169 574	423 483	1 118 178	538 151	1 740 523
2026	743	663	839	43 809	39 366	48 436	312 599	180 908	452 785	1 190 297	572 461	1 853 986
2027	793	707	894	46 707	41 953	51 660	333 279	192 796	482 923	1 260 999	605 970	1 965 604
2028	840	748	949	49 505	44 422	54 803	353 243	204 142	512 311	1 332 238	639 766	2 077 974
2029	889	792	1 005	52 390	46 992	58 018	373 828	215 952	542 363	1 402 790	673 164	2 189 472
2030	937	833	1 059	55 206	49 477	61 182	393 920	227 374	571 934	1 473 849	706 824	2 301 712
2031	986	877	1 115	58 086	52 039	64 396	414 473	239 146	601 984	1 544 096	740 040	2 412 843

Technical Appendix Table 5. Predicted incremental avoided health outcomes from 2012–2031 for scenario C with 90% vaccination coverage and a mean duration of 2 y of complete vaccine protection

	Cumulative avoided deaths			Cumulative avoided hospitalizations			Cumulative avoided outpatient visits			Cumulative avoided homecare episodes		
Year	C	Low	High	C	Low	High	C	Low	High	C	Low	High
2012	24	22	26	1 407	1 311	1 504	10 042	6 026	14 056	42 593	21 266	63 982
2013	70	65	77	4 149	3 850	4 451	29 605	17 693	41 604	127 686	63 576	192 335
2014	130	120	143	7 679	7 122	8 242	54 793	32 727	77 045	232 402	115 377	351 092
2015	179	164	198	10 552	9 713	11 408	75 295	44 636	106 642	324 443	160 316	492 421
2016	234	214	259	13 798	12 681	14 939	98 454	58 274	139 650	417 173	205 435	635 281
2017	282	256	313	16 618	15 193	18 082	118 578	69 818	169 031	503 492	247 092	769 296
2018	333	301	370	19 605	17 890	21 369	139 890	82 213	199 762	587 580	287 631	899 976
2019	380	343	424	22 398	20 387	24 472	159 824	93 689	228 772	669 164	326 942	1 026 828
2020	427	385	477	25 153	22 857	27 524	179 481	105 040	257 303	747 176	364 468	1 148 316
2021	473	426	528	27 845	25 270	30 507	198 687	116 127	285 184	823 119	401 010	1 266 551
2022	517	465	579	30 463	27 613	33 413	217 372	126 897	312 348	896 776	436 426	1 381 301
2023	561	504	628	33 057	29 938	36 286	235 877	137 582	339 206	968 528	470 920	1 493 105
2024	604	543	677	35 616	32 229	39 124	254 135	148 110	365 739	1 039 316	504 951	1 603 405
2025	649	582	727	38 225	34 569	42 015	272 755	158 863	392 761	1 110 564	539 213	1 714 392
2026	694	622	778	40 876	36 944	44 953	291 668	169 775	420 231	1 182 773	573 940	1 826 869
2027	739	662	829	43 538	39 328	47 906	310 667	180 733	447 834	1 255 041	608 673	1 939 501
2028	784	702	880	46 189	41 701	50 848	329 583	191 637	475 335	1 327 135	643 302	2 051 926
2029	829	742	931	48 845	44 077	53 796	348 533	202 555	502 891	1 399 497	678 050	2 164 797
2030	874	783	983	51 505	46 456	56 749	367 514	213 489	530 501	1 471 926	712 816	2 277 818
2031	919	823	1 034	54 163	48 832	59 701	386 479	224 410	558 096	1 544 203	747 494	2 390 646

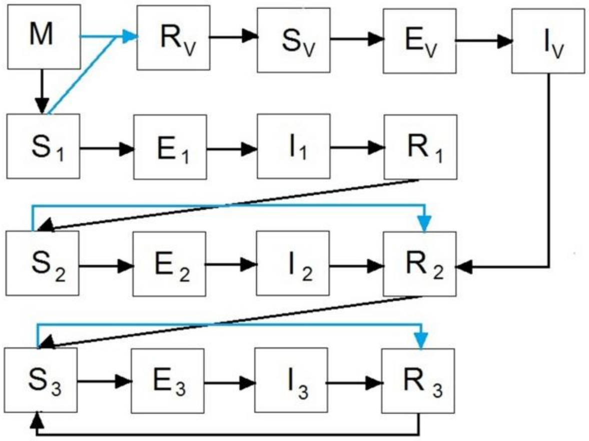

Technical Appendix Figure 1. Model schematics of the SEIR dynamic natural history model. The effect of vaccination is shown with blue arrows.

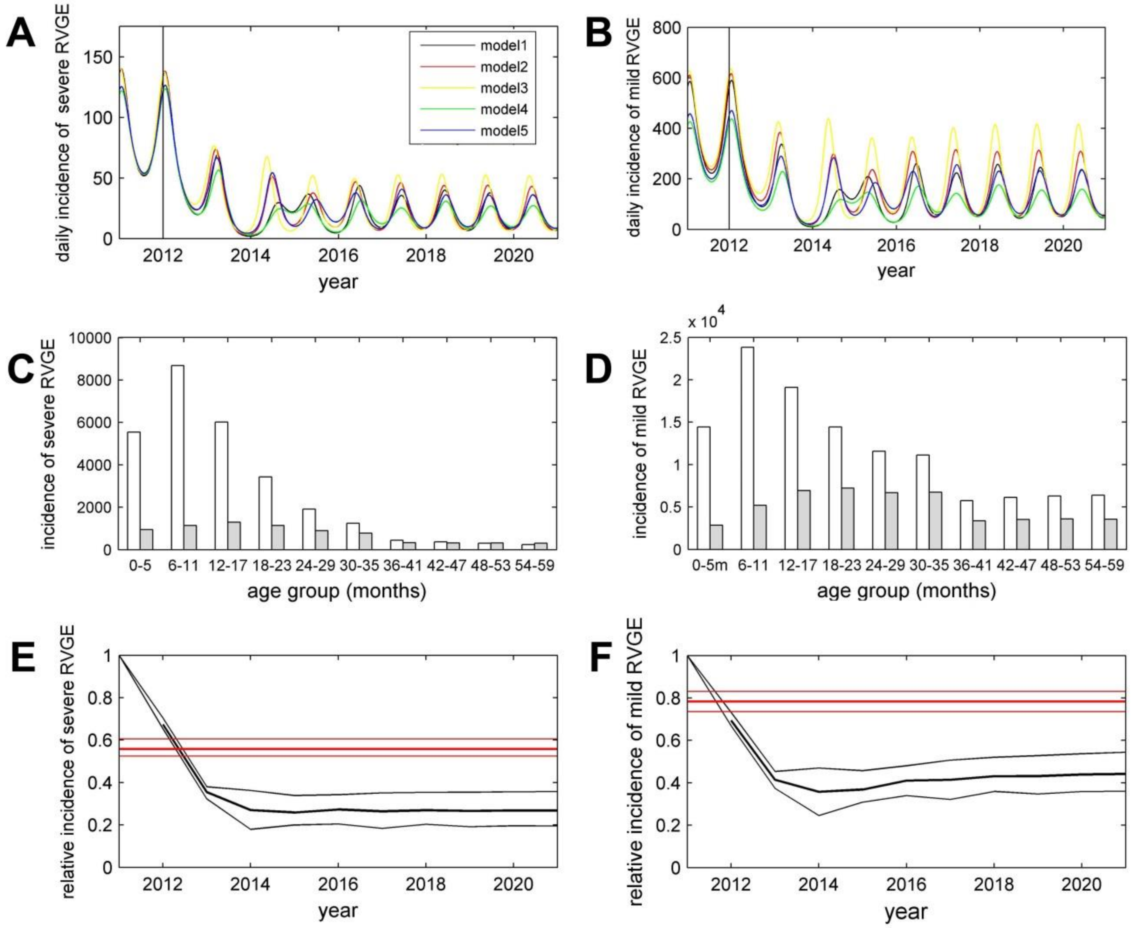

Technical Appendix Figure 2. Model fitting to sentinel hospitalization data (N = 1013 weeks) from Kazakhstan, 2007–2009; A) Age-specific model in 2-month age groups; B) Weekly incidence of hospitalizations caused by rotavirus (solid line) and fitted incidence of severe infections in the model (dashed line); the total numbers of severe infections in children <5 years were multiplied with a factor $p\_sev = 0.01556$ to yield a similar mean as for the observational data. *GE: gastroenteritis.

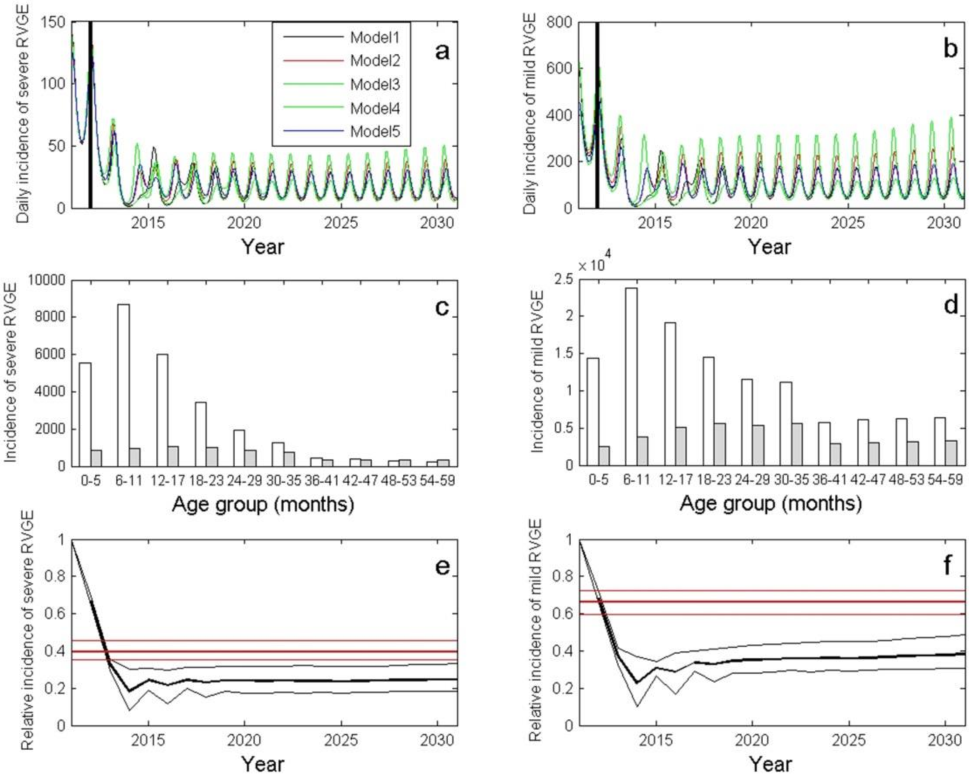

Technical Appendix Figure 3. Epidemiologic impact of rotavirus vaccination in children <5 years in Kazakhstan with a 2-year mean vaccine protection. A) Estimated daily incidence of severe RVGE (base case) with introduction of rotavirus vaccination in January 2012 in the five candidate models; B) Estimated daily incidence of mild RVGE (base case) with introduction of the rotavirus vaccination in January 2012 in the five candidate models using a similar color scheme as shown in legend A); C) Yearly age-specific incidence of severe RVGE pre-vaccination (white) and 10 years post-vaccination (gray); Yearly age-specific incidence of mild RVGE pre-vaccination (white) and 10 years post-vaccination (gray); E) Relative incidence of severe RVGE with vaccination compared with the expected incidence without vaccination; the blue curve shows the mean relative incidence with lower and upper bounds predicted by the synthesis of dynamic models, including both direct and indirect effects, while the red curve shows the relative incidence predicted from a static cohort model incorporating only the direct effects (Supplemental Section S.3); F) Relative incidence of mild RVGE with vaccination compared with the expected incidence without vaccination; the blue curve shows the mean relative incidence with lower and upper bounds in the synthesis of dynamic models, while the red curve shows the relative incidence predicted by a static cohort model.

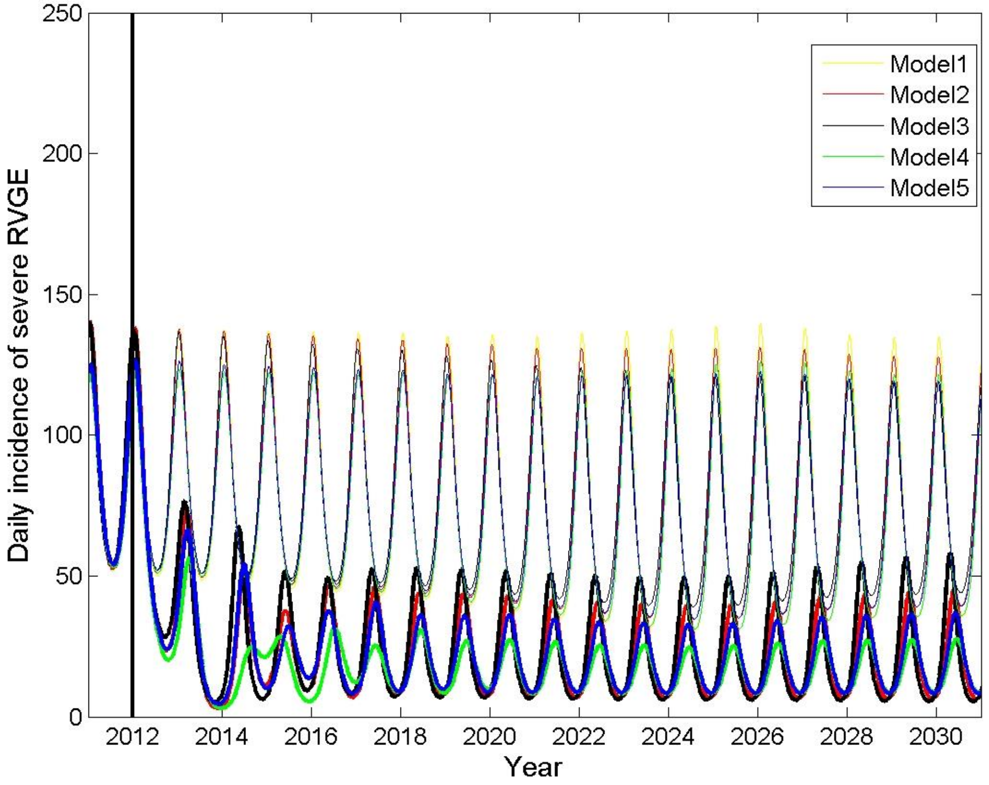

Technical Appendix Figure 4. Epidemiologic effects of rotavirus vaccination in children <5 years in base case model: Estimated daily incidence of severe rotavirus gastroenteritis in the 5 candidate models with introduction of rotavirus vaccination in January 2012 (fat lines) and without vaccination implemented (slim lines).
